# Genomic Analyses of the Quinol Oxidases and/or Quinone Reductases Involved in Bacterial Extracellular Electron Transfer

**DOI:** 10.3389/fmicb.2018.03029

**Published:** 2018-12-10

**Authors:** Yuhong Zhong, Liang Shi

**Affiliations:** ^1^Department of Biological Sciences and Technology, School of Environmental Studies, China University of Geosciences, Wuhan, China; ^2^State Key Laboratory of Biogeology and Environmental Geology, China University of Geosciences, Wuhan, China

**Keywords:** quinol oxidase, quinone reductase, multiheme *c*-type cytochromes, extracellular electron transfer pathways, the cytoplasmic membrane

## Abstract

To exchange electrons with extracellular substrates, some microorganisms employ extracellular electron transfer (EET) pathways that physically connect extracellular redox reactions to intracellular metabolic activity. These pathways are made of redox and structural proteins that work cooperatively to transfer electrons between extracellular substrates and the cytoplasmic membrane. Crucial to the bacterial and archaeal EET pathways are the quinol oxidases and/or quinone reductases in the cytoplasmic membrane where they recycle the quinone/quinol pool in the cytoplasmic membrane during EET reaction. Up to date, three different families of quinol oxidases and/or quinone reductases involved in bacterial EET have been discovered. They are the CymA, CbcL/MtrH/MtoC, and ImcH families of quinol oxidases and/or quinone reductases that are all multiheme *c*-type cytochromes (*c*-Cyts). To investigate to what extent they are distributed among microorganisms, we search the bacterial as well as archaeal genomes for the homologs of these *c*-Cyts. Search results reveal that the homologs of these *c*-Cyts are only found in the Domain Bacteria. Moreover, the CymA homologs are only found in the phylum of Proteobacteria and most of them are in the *Shewanella* genus. In addition to *Shewanella* sp., CymA homologs are also found in other Fe(III)-reducing bacteria, such as of *Vibrio parahaemolyticus*. In contrast to CymA, CbcL/MtrH/MtoC, and ImcH homologs are much more widespread. CbcL/MtrH/MtoC homologs are found in 15 phyla, while ImcH homologs are found in 12 phyla. Furthermore, the heme-binding motifs of CbcL/MtrH/MtoC and ImcH homologs vary greatly, ranging from 3 to 23 and 6 to 10 heme-binding motifs for CbcL/MtrH/MtoC and ImcH homologs, respectively. Moreover, CymA and CbcL/MtrH/MtoC homologs are found in both Fe(III)-reducing and Fe(II)-oxidizing bacteria, suggesting that these families of *c*-Cyts catalyze both quinol-oxidizing and quinone-reducing reactions. ImcH homologs are only found in the Fe(III)-reducing bacteria, implying that they are only the quinol oxidases. Finally, some bacteria have the homologs of two different families of *c*-Cyts, which may improve the bacterial capability to exchange electrons with extracellular substrates.

## Introduction

Many microorganisms can exchange electrons between the redox proteins in the cytoplasmic or inner membrane and extracellular substrates, such as metal ions associated with minerals, humic substances and electrodes, and microbial cells of the same or difference species. For example, the metal-reducing bacteria *Geobacter* spp. and *Shewanella* spp. use solid phase Fe(III)-containing minerals, humic substances and electrodes as terminal electron acceptors for anaerobic respiration (Lovley et al., 1987, 1996; Myers and Nealson, 1988; Bond and Lovley, 2003; Bretschger et al., 2007). The metal-oxidizing bacteria *Mariprofundus ferrooxydans* PV-1 and *Rhodopseudomonas palustris* TIE-1 use Fe(II) and electrodes as electron and/or energy source for growth (Jiao et al., 2005; Emerson et al., 2007; Summers et al., 2013; Bose et al., 2014). Moreover, *Geobacter metallireducens* transfers electrons directly to *Geobacter sulfurreducens* via conductive nanowires and multiheme *c*-type cytochromes (*c*-Cyts) (Summers et al., 2010). *Shewanella oneidensis* MR-1 transfers electrons from one cell to another via the multiheme *c*-Cyts on the outer membrane extension that connects the two cells (Gorby et al., 2006; Pirbadian et al., 2014; Subramanian et al., 2018). Electron exchange between the microbial cytoplasmic membrane and extracellular substrates or cells is often referred to as microbial extracellular electron transfer (EET) (Shi et al., 2016).

To exchange electrons extracellularly, some microorganisms form EET pathways that consist of structural and redox proteins. For example, *S. oneidensis* MR-1 possesses a metal-reducing (Mtr) EET pathway. The currently identified protein components of the Mtr pathway include six multiheme *c*-Cyts: the cytoplasmic membrane CymA, the periplasmic Fcc_3_ and STC and the outer membrane MtrA, MtrC and OmcA, and one outer membrane porin protein MtrB (Shi et al., 2016). The Mtr pathway starts at CymA that oxidizes the quinol in the cytoplasmic membrane (Marritt et al., 2012a,b; McMillan et al., 2012, 2013). The released electrons are transferred first through the periplasm by Fcc_3_ and STC (Firer-Sherwood et al., 2011; Fonseca et al., 2013; McMillan et al., 2013) and then across the outer membrane to the bacterial surface by MtrABC (Hartshorne et al., 2009; White et al., 2012, 2013). On the bacterial surface, OmcA interacts with MtrC to mediate the electron transfer to Fe(III)-containing minerals (Shi et al., 2006; Zhang et al., 2008, 2009). To date, the Mtr pathway of *S. oneidensis* MR-1 is the most rigorously characterized microbial EET pathway (Shi et al., 2016). Similar to the Mtr pathway, the porin-cytochrome (Pcc) EET pathways are proposed in *G. sulfurreducens*, which also includes multiheme *c*-Cyts and the outer membrane porin proteins (Shi et al., 2016). In the Pcc pathways, the porin-cytochrome protein complexes for transferring electrons across the outer membrane to the Fe(III)-containing minerals are functionally verified (Liu et al., 2014). Although they are biochemically and biophysically characterized (Lloyd et al., 2003; Morgado et al., 2010), the proposed EET role of the periplasmic multiheme *c*-Cyts PpcA and PpcD remain to be demonstrated. In addition, the proposed cytoplasmic membrane quinol oxidases CbcL and ImcH also remain to be characterized. The Mtr and Pcc pathways transfer electrons from the CymA, CbcL and ImcH in the cytoplasmic membrane to the extracellular Fe(III)-containing minerals, in which the CymA, CbcL, and ImcH sever or are proposed to sever as quinol oxidases. The metal-oxidizing (Mto) pathway of the Fe(II)-oxidizing bacterium *Sideroxydans lithotrophicus* ES-1 is, however, proposed to transfer electrons from extracellular electron donors to CymA in the cytoplasmic membrane where CymA is proposed to function as a quinone reductase (Liu et al., 2012; Shi et al., 2012). Thus, CymA, CbcL, and ImcH play or are proposed to play crucial roles in redox cycling of quinone/quinol pool in the cytoplasmic membrane during bacterial EET.

**FIGURE 1:**
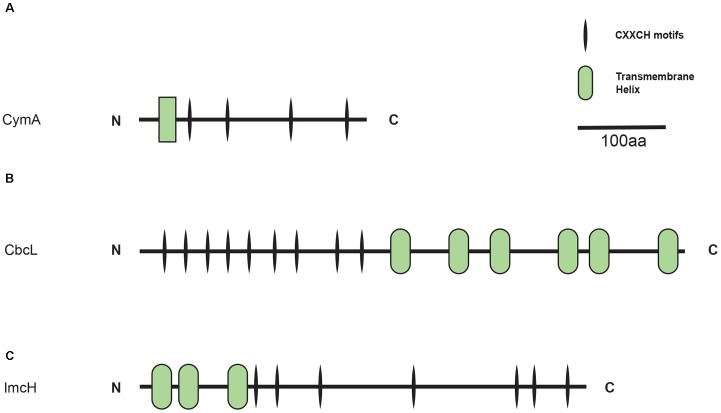
Schematic representation of transmembrane and CX_2_CH (i.e., heme-binding) motifs of CymA of *Shewanella oneidensis* MR-1 **(A)**, CbcL of *Geobacter sulfurreducens* PCA **(B)**, and ImcH of *G. sulfurreducens* PCA **(C)**. C, *C*-terminus; N, *N*-terminus; aa, amino acid residues.

CymA, CbcL, and ImcH belong to different protein families and each exhibits its own characteristics. For example, as a member of the NapC/NirT family of quinol dehydrogenases, CymA of *S. oneidensis* MR-1 is a tetra-heme *c*-Cyt that possesses a short transmembrane domain at its *N*-terminus and a periplasmic domain at its *C*-terminal region (Figure 1A; Shi et al., 2007). Different from CymA, CbcL of *G. sulfurreducens* is predicted to have nine heme-binding motifs (CX_2_CH) of *c*-Cyts in its N-terminal half and six transmembrane helices in its C-terminal half (Figure 1B). The transmembrane region of CbcL also displays a high degree of similarity to the HydC/FdnI-like *b*-type cytochromes that contain two putative hemes (Zacharoff et al., 2015). Finally, ImcH of *G. sulfurreducens* is predicted to possess three transmembrane helices in its *N*-terminal region and seven CX_2_CH motifs in its *C*-terminal region (Figure 1C). It should be pointed out that part of ImcH, which includes the third transmembrane helix and the first three CX_2_CH motifs adjacent to the third helix, show a certain degree of similarity to the NapC/NirT family of quinol dehydrogenases (Levar et al., 2014).

In addition to *S. oneidensis* MR-1 and *S. lithotrophicus* ES-1, CymA homologs also exist in other metal-reducing bacteria, such as *Shewanella* sp. strain ANA-3, *Shewanella putrefaciens* W3-18-1, *Ferrimonas balearica* and *Rhodoferax ferrireducens* (Murphy and Saltikov, 2007; Shi et al., 2012; Wei et al., 2016). Similarly, MtoC and MtrH, which are the homologs of CbcL, are also present in *R. ferrireducens* as well as the metal-oxidizing bacteria *Dechloromonas aromatica* RCB and *Gallionella capsiferriformans* ES-2 (Shi et al., 2012). Moreover, in *R. ferrireducens*, two *cymA* genes and a *mtrH* gene are found in the same gene cluster that also contains *mtrABC* genes and other *c*-Cyts-encoding genes (Shi et al., 2012). Given that they are found in both metal-reducing and metal-oxidizing bacteria, CbcL/MtoC/MtrH homologs are most likely involved in both quinol oxidation as well as quinone reduction. However, distribution of CymA and CbcL/MtoC/MtrH homologs in other bacteria have not been systemically investigated. Different from that from the homologs of CymA and CbcL/MtoC/MtrH, distribution of ImcH homologs in different bacteria has been searched. Search results showed that, in addition to *Geobacter* spp. ImcH homologs are found in a variety of other bacteria, including the Fe(III)-reducing bacteria *Acidobacterium capsulatum*, *Anaeromxyobacter* 2PC-C*, Geoalkalibacter subterraneus*, *Geothrix fermantans*, and *Melioribacter roseus*, where they are proposed to function as quinol oxidases (Levar et al., 2014; Shi et al., 2016). However, it remains unclear whether, similar to CymA and CbcL/MtoC/MtrH homologs, ImcH homologs also exist in the metal-oxidizing bacteria. Thus, in this study, we systematically searched the bacterial and archaeal genomes available in May 16, 2018 for the homologs of CymA, CbcL/MtoC/MtrH and ImcH to address the following questions: (1) To what extent CymA, CbcL/MtoC/MtrH and ImcH homologs are distributed among the sequenced microorganisms? (2) Is ImcH homolog also present in the metal-oxidizing bacteria?

### Approach

#### Search for the CymA, MtrH/MtoC/CbcL and ImcH Homologs

The bacterial and archaeal genomes were searched for CymA, CbcL/MtoC/MtrH and ImcH homologs by the approach described before (Shi et al., 1998, 2012, 2014; Shi and Zhang, 2004). In the beginning, previously identified CymA homologs of *F. balearica*, *R. ferrireducens, S. lithotrophicus* ES-1 and *S. oneidensis* MR-1, CbcL/MtoC/MtrH homologs of *D. aromatica* RCB, *G capsiferriformans* ES-2, *G. sulfurreducens* and *R. ferrireducens*, as well as ImcH homologs of *A. capsulatum*, *A.* 2PC-C*, G. subterraneus*, *G. sulfurreducens, G. fermantans*, and *M. roseus* were used as templates to search for the open reading frames (ORFs) whose deduced polypeptide sequences exhibited similarity to the templates by BLAST (*E* < 0.01) (Altschul et al., 1990; Supplementary Tables S1–S3). The search was carried by using the blastp program of the National Center for Biotechnology Information (NCBI) and the BLAST program of the Universal Protein Resource (UniProt). The search parameters are: scoring matrix = BLOSUM62, gapopen = 0, and gapextend = 0. The databases searched were non-redundant protein sequences database (nr) and UniprotKB database. The tentatively identified homologs were subject to inspection with in-house Perl scripts for the CX_2_CH motifs as well as the analyses by a hidden Markov model-based TMHMM software for predicting the transmembrane helices (Krogh et al., 2001; Shi et al., 2012, 2014). The verified homologs were also used as templates for next round of genome search. Additionally, the ORFs adjacent to that of the verified homologs were also searched for those encoding putative *c*-Cyts (Shi et al., 2012, 2014).

#### Phylogenetic Reconstruction and Gene Cluster Identification

The polypeptide sequences were aligned by Clustal W (version 2.1) with the following settings: Gap Opening Penalty = 10; Gap Extension Penalty = 0.2; Protein matrix = BLOSUM series (Larkin et al., 2007). The aligned sequences of all verified CymA, CbcL/MtoC/MtrH or ImcH homologs were analyzed separately by MEGA7. Maximum Likelihood method was used to construct phylogenetic trees with a confidence level determined by 1000 bootstrap replications (Kumar et al., 2016). The reconstructions were performed by applying the best amino acid model of amino acid substitutions, which was determined by Close-Neighbor-Interchange model (Kumar et al., 2016). The Evolview v2 was used to graphically display the results of phylogenetic reconstruction (He et al., 2016). After the homologs were acquired, other genes, whose polypeptides had CX_2_CH motifs on upstream and downstream of the homologs, were also identified by the method described above. The figure was plotted by IBS (Liu et al., 2015).

## Results and Discussion

### Overview

As shown in Table 1, a total of 282 homologs were identified, which include 73 CymA homologs, 138 CbcL/MtoC/MtrH homologs, and 71 ImcH homologs. All of them are in the Domain Bacteria. Sixty-three percent of identified CymA homologs were found in the genus of *Shewanella* and the rest were found in 11 bacterial genera and 2 bacteria that were not classified at the genus level, which were all in the phylum of Proteobacteria (Table 1 and Supplementary Table S1). Compared to the CymA homologs that are found only one phylum, CbcL/MtoC/MtrH homologs were much more widespread and were identified in 15 bacterial phyla that included 33 genera and the bacteria whose classification could not be assigned to the genus level. Seventeen CbcL/MtoC/MtrH homologs were found in the genus of *Geobacter* (Table 1 and Supplementary Table S2). Similar to the CbcL/MtoC/MtrH homologs, ImcH homologs were also widespread and were found in 12 phyla, which included 19 genera and the bacteria not classified to the genus level. In the genus of *Geobacter*, 18 ImcH homologs were identified.(Table 1 and Supplementary Table S3).

**Table 1:** Summary of identified CymA, CbcL/MtoC/MtrH, and ImcH homologs in different bacterial phyla.

Phyla	CymA	CbcL/MtoC/MtrH	ImcH
Proteobacteria	73^a^	103	36
Acidobacteria	0	2	19
Actinobacteria	0	0	1
Bacteroidetes	0	5	1
Calditrichaeota	0	1	1
Chloroflexi	0	0	1
Elusimicrobia	0	2	0
Gemmatimonadetes	0	1	2
Ignavibacteriae	0	2	3
Lentisphaerae	0	1	0
Nitrospirae	0	1	0
Planctomycetes	0	2	0
Verrucomicrobia	0	4	1
Candidatus Dadabacteria	0	1	0
Candidatus Handelsmanbacteria	0	1	0
Candidatus Omnitrophica	0	3	3
Candidatus Rokubacteria	0	0	1
Candidate Phylum	0	2	0
Others^b^	1	7	2

^a^*The number indicating the numbers of homologs identified in that phylum. ^*b*^The number indicating the numbers of homologs in the bacteria that could not be classified to the phylum level*.

### The CymA Homologs

**FIGURE 2:**
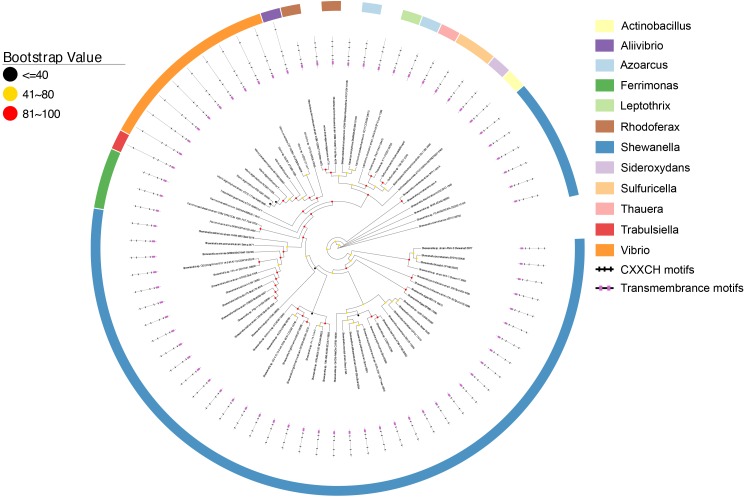
Distribution of CymA homologs. Inner layer, phylogenetic analysis of identified CymA homologs. A phylogenetic tree was constructed and graphically displayed as described in the text. Middle layer, schematic representation of length of and positions of heme-binding as well as transmembrane motifs in the polypeptides of the CymA homologs. Outer layer, bacterial genera from which CymA homologs were identified.

As shown in Figure 2, all CymA homologs of *Shewanella* sp. were clustered together and they were 58.8–100% identical. This observation is consistent with previous findings (Murphy and Saltikov, 2007) and suggests that the functional role of CymA homologs is well conserved in *Shewanella* sp. The identity between the CymA homologs of *Shewanella* sp. and those identified from other bacteria ranged from 22.5 to 96.7%. The polypeptides of nearly all CymA homologs from *Shewanella* sp. contained 187 amino acid residues, except those of *Shewanella* sp. GutDb and *S. piezotolerans*strain WP3 whose polypeptides possessed186 and 188 amino acid residues, respectively (Figure 2 and Supplementary Table S1). The polypeptides of the CymA homologs from other bacteria were 187 to 364 amino acid residue long. Notably, the polypeptides of CymA homologs from 9 *Vibrio* sp., *Aliivibrio* sp. 1S175 and *Trabulsiella guamensis* had 363–364 amino acid residues, which were much longer than those of the rest of identified CymA homologs (Figure 2 and Supplementary Table S1). Moreover, compared to those with much shorter polypeptides, the CymA homologs with longer polypeptides possessed an extra CX_2_CH motif close to their C-termini (Figure 2 and Supplementary Table S1). Finally, a putative atypical heme-binding motif CX_3_CH and AX_2_CH was found in the polypeptide sequence close to the transmembrane domain for the two CymA homologs from *R. ferrireducens* and the one identified from the metagenome of a mine drainage, respectively (Shi et al., 2012). Thus, all identified CymA homologs contained 4 heme-binding motifs, except those from *Vibrio* sp., *Aliivibrio* sp. 1S175 and *T. guamensis*, which contained 5 heme-binding motifs (Figure 2 and Supplementary Table S1).

**FIGURE 3:**
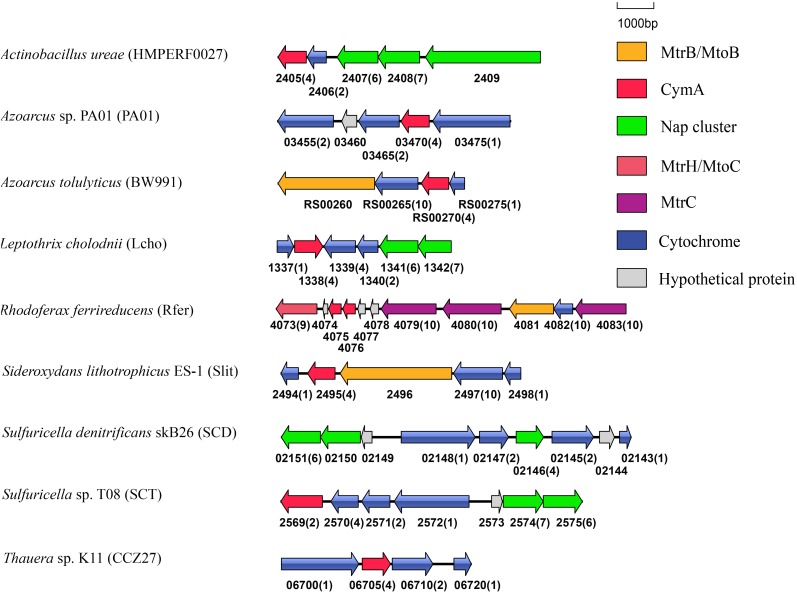
Genetic organization of *cymA* and its associated genes encoding *c*-Cyts and MtrB/MtoB. Shown are the relative positions of genes identified within the complete nucleotide sequences of these bacteria. The genes are labeled by arrows whose sizes and orientations indicate their relative lengths and directions in which they are presumed to be transcribed. The numbers displayed below the gene clusters are part of their locus tags whose letter parts are displayed in the parenthesis after species name. The numbers in parenthesis after the locus tag indicating the numbers of heme-binding motifs in the polypeptides deduced from *c*-Cyt-encoding genes.

In addition to *Shewanella* sp., CymA homologs were identified from the bacteria that reduced solid phase Fe(III)-containing and/or Mn(IV)-containing minerals, such as *F. balearica, R. ferrireducens*, and *Vibrio parahaemolyticus* and the bacteria that oxidized Fe(II) or Mn(II), such as *Sideroxydans lithotrophicus* ES-1 and *Leptothrix cholodnii* (Emerson and Moyer, 1997; Finneran et al., 2003; Nolan et al., 2010; Takeda et al., 2012; Wee et al., 2014). Previous results showed that the *cymA* homologs of *F. balearica, R. ferrireducens*, and *S. lithotrophicus* ES-1 were part of gene clusters that also contained genes whose homologs were involved in extracellular electron transfer (Shi et al., 2012; Emerson et al., 2013; Figure 3). Further analysis of genes adjacent to the *cymA* genes revealed that, like the cymA found in *F. balearica, R. ferrireducens*, and *S. lithotrophicus*, *a mtrB/mtoB*-like gene and a *mtrA*/*mtoA*-like gene were also next to the *cymA* gene of *Azoarcus tolulyticus* (Figure 3). Thus, the CymA homologs found in these bacteria are most likely involved in EET. Furthermore, *mtrABC* homologs were also found in *V. parahaemolyticus* in which they participated in extracellular reduction of Fe(III)-containing and Mn(IV)-containing minerals (Shi et al., 2012; Wee et al., 2014). Although it is not part of the *mtrABC* gene cluster, which is similar to that in *Shewanella* sp., the *cymA* homolog of *V. parahaemolyticus*is also likely involved in extracellular reduction of Fe(III)-containing and Mn(IV)-containing minerals. Given that *cymA* and *mtrABC* homologs are found in several *Vibrio* sp. (Shi et al., 2012), EET may also be widespread in *Vibrio* sp. *L. cholodnii* oxidizes Mn(II) extracellularly (Takeda et al., 2012). However, whether the released electrons after Mn(II) oxidation are transferred inside bacterial cells remains unknown. Therefore, the role of CymA homolog of *L. cholodnii* in EET is also unclear.

CymA is a member of the NapC/NirT family of quinol dehydrogenases (Shi et al., 2007). In *S. oneidensis* MR-1, CymA is involved in extracellular reduction of Fe(III)- and Mn(IV)-containing minerals and DMSO as well as in reduction of fumarate, nitrate and nitrite in the periplasm (Myers and Myers, 1997, 2000; Schwalb et al., 2003; Lies et al., 2005; Gralnick et al., 2006). Furthermore, in *Shewanella* sp. strain ANA-3 and *S. putrefaciens* strain CN-32, CymA is involved in arsenate reduction (Murphy and Saltikov, 2007). Notably, *cymA* genes were often found next to the *napG* and *napH* genes that are involved in nitrate reduction (Figure 3; Richardson, 2000). The CymA homologs found in these bacteria, such as *Actinobacillus ureae*, *Leptothrix cholodnii*, *Sulfuricella* sp. T08 and *Sulfuricella denitrificans* skB26 are probably also involved in nitrate reduction. Finally, in *Azoarcu*s sp. PA01 and *Thauera* sp. K11, *cymA* genes were also found next to the genes encoding putative multi-heme *c*-Cyts without defined functions (Figure 3).

Except the CymA homologs of *S. lithotrophicus* ES-1, no additional CymA homolog was found in the Fe(II)-oxidizing bacteria in this study. In *S. lithotrophicus* ES-1, the CymA homolog was proposed to function as a quinone reductase (Liu et al., 2012; Shi et al., 2012, 2016). Lack of additional CymA homologs from the metal-oxidizing bacteria renders it impossible to assess the difference between CymA of the metal-oxidizing bacteria with those of the metal-reducing bacteria. CymA of *S. lithotrophicus* ES-1 was 33.1% identical to CymA of *S. oneidensis* MR-1, which is a functional quinol oxidase (Marritt et al., 2012a,b; McMillan et al., 2012, 2013). The identities between CymA of *S. lithotrophicus* ES-1 and CymA homologs of other bacteria ranged from 29.4 to 66.4%. CymA of *S. oneidensis* MR-1 possesses intrinsic quinone reductase activty and its quinol oxidase activitiy is activated by its physical association with the periplasmic *c*-Cyt Fcc_3_ (McMillan et al., 2013). Thus, the catalytic activity and electron transfer direction of CymA of *S. oneidensis* MR-1 and probably the CymA homologs in other bacteria are regulated by their interaction with other proteins.

### The CbcL/MtoC/MtrH Homologs

**FIGURE 4:**
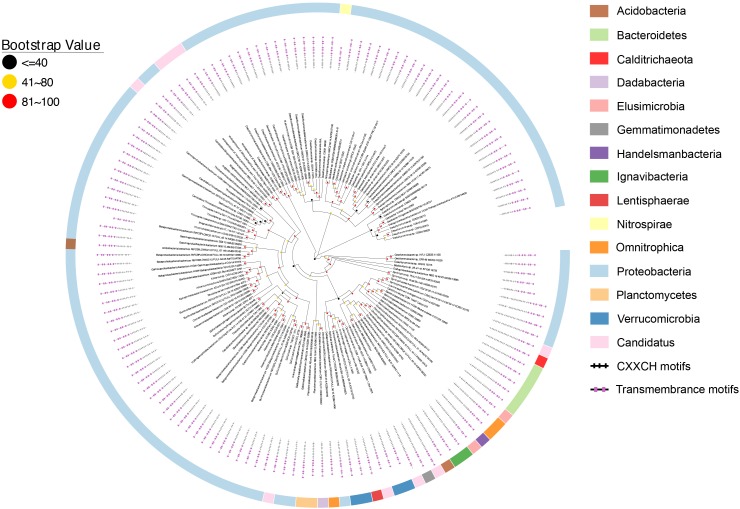
Distribution of CbcL/MtrH/MtoA homologs. Inner layer, phylogenetic analysis of identified CbcL/MtrH/MtoA homologs. A phylogenetic tree was constructed and graphically displayed as described in the text. Middle layer, schematic representation of length of and positions of heme-binding as well as transmembrane motifs in the polypeptides of the CbcL/MtrH/MtoA homologs. Outer layer, bacterial phyla from which CbcL/MtrH/MtoA homologs were identified.

Figure 4 shows that CbcL homologs of *Geobacter* sp. are clustered together as well as with the homologs from other bacteria, such as *Desulfuromonas* sp. and *Syntrophus* sp. The identity among these homologs ranged from 57.6 to 100%, demonstrating the conserved functional roles of CbcL homologs in these bacteria. MtoC of the Fe(II)-oxidizing bacterium *D. aromatica* strain RCB was clustered with those identified from metagenomic sequencing the samples collected from underground water at the Horonobe Underground Research Laboratory (URL) (Shi et al., 2012). The identity among these homologs were 63.2–82.7%. As no apparent Fe(II)-oxidizing bacterium was found in the same site (Hernsdorf et al., 2017), the roles of MtoC homologs clustered with that of *D. aromatica* strain RCB remain uncertain. Additionally, MtoC of the Fe(II)-oxidizing bacterium *G. capsiferriformans* strain ES-2 was clustered with ones of a bacterium from *Gallionellales*, the sulfur-oxidizing bacterium *Sulfuricaulis limicola* and a bacterial community from a pilot plant for the treatment of acid mine drainage (AMD) from the lignite mining district in Lusatia, Germany. They were 58.2–91.9% identical. Notably, the MtoC homologs of *D. aromatica* strain RCB and *G. capsiferriformans* strain ES-2 were only 58.7% identical. Moreover, MtrH of the Fe(III)-reducing bacterium *R. ferrireducens* was clustered with the homologs of the bacteria with no apparent role in EET and was 55.4 and 55.2% identical to MtoCs of *G. capsiferriformans* strain ES-2 and *D. aromatica*, respectively. Finally, the identities between CbcL of *G. sulfurreducens* and MtrH of *R. ferrireducens* as well as MtoCs of *D. aromatica* RCB and *G. capsiferriformans* ES-2 were 35.7, 35.4, and 35.5%, respectively.

The overall identities among identified CbcL/MtrH/MtoC homologs ranged from 28.6 to 100%. The polypeptide length of identified CbcL/MtrH/MtoC homologs ranged from 416 to 1034 amino acid residues (Figure 4 and Supplementary Table S2). In addition, their *N*-terminal portions also varied substantially, of which the CX_2_CH motifs ranged from 3 to 23 (Figure 4 and Supplementary Table S2). Moreover, most identified CbcL/MtrH/MtoC homologs are predicted to possess six transmembrane motifs. However, four homologs whose accession numbers are PKN12990.1, PLY03765.1, OGT98815.1, and OQX95005.1, respectively, have five transmembrane motifs and one homolog with an accession number of OGU12690.1 contains only four transmembrane motifs (Figure 4 and Supplementary Table S2). Finally, the three histidine residues that might be the ligands for *b*-type hemes were conserved in the transmembrane regions.

**FIGURE 5:**
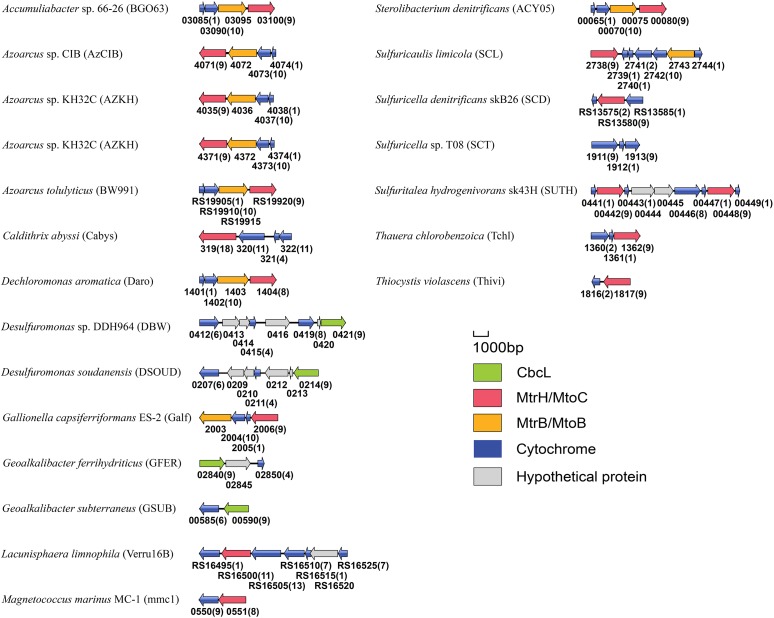
Genetic organization of *cbcL*, *mtrH*, *mtoC* and their associated genes encoding *c*-Cyts and MtrB/MtoB. Shown are the relative positions of genes identified within the complete nucleotide sequences of these bacteria. The genes are labeled by arrows whose sizes and orientations indicate their relative lengths and directions in which they are presumed to be transcribed. The numbers displayed below the gene clusters are part of their locus tags whose letter parts are displayed in the parenthesis after species name. The numbers in parenthesis after the locus tag indicating the numbers of heme-binding motifs in the polypeptides deduced from *c*-Cyt-encoding genes.

Similar to *cymA*, *mtrH* and *mtoC* genes are often associated with other *c*-Cyt genes. For instance, in the genome of *R. ferrireducens*, *mtrH* is part of a gene cluster that also contains *cymA*, *mtrA*, *mtrB*, *mtrC*, and *mtrH* (Figure 5). In the genomes of *D. aromatica* RCB and *G. capsiferriformans*, *mtoC* is in a gene cluster that contains *mtoA*, *mtoB*, and *mtoD* (Figure 5; Shi et al., 2012). Our results from this study showed that *mtrH*/*mtoC* genes of 7 bacterial species were also part of a gene cluster containing *mtrAB*/*mtoAB* genes (Figure 5). Given that *mtrAB*/*mtoAB* genes function to transfer electrons across the outer membrane (Hartshorne et al., 2009; Richardson et al., 2012; Shi et al., 2012), the *mtrH*/*mtoC* genes found with *mtrAB*/*mtoAB* genes are most likely involved in EET. Moreover, the *cbcL*/*mtrH*/*mtoC* genes of other 11 bacterial species were also adjacent to *c*-Cyt-encoding genes (Figure 5). Further comparisons between CbcL of *G. sulfurreducens*/MtrH of *R. ferrireducens* with MtoC of *D. aromatica* RCB and *G. capsiferriformans* ES-2, however, did not reveal any unique feature for those catalyzing quinol oxidation or quinone reduction.

### The ImcH Homologs

**FIGURE 6:**
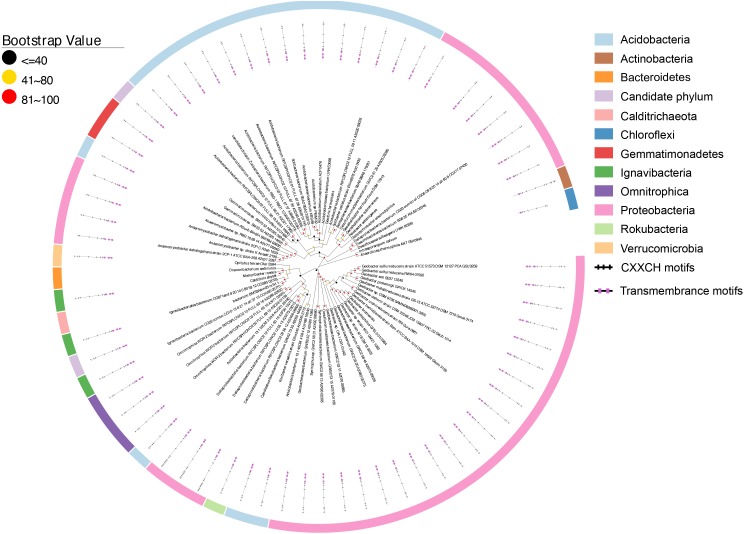
Distribution of ImcH homologs. Inner layer, phylogenetic analysis of identified ImcH homologs. A phylogenetic tree was constructed and graphically displayed as described in the text. Middle layer, schematic representation of length of and positions of heme-binding as well as transmembrane motifs in the polypeptides of the ImcH homologs. Outer layer, bacteria phyla from which ImcH homologs were identified.

**FIGURE 7:**
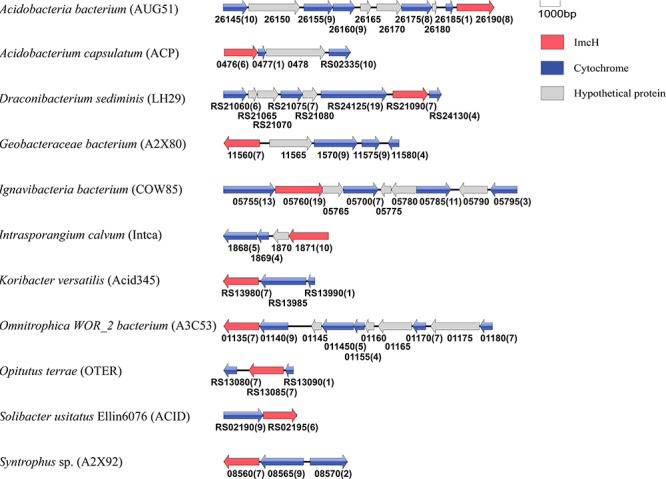
Genetic organization of *imcH* and its associated genes encoding *c*-Cyts. Shown are the relative positions of genes identified within the complete nucleotide sequence of these bacteria. The genes are labeled by arrows whose sizes and orientations indicate their relative lengths and directions in which they are presumed to be transcribed. The numbers displayed below the gene clusters are part of their locus tags whose letter parts are displayed in the parenthesis after species name. The numbers in parenthesis after the locus tag indicating the numbers of heme-binding motifs in the polypeptides deduced from *c*-Cyt-encoding genes.

Similar to the CbcL homologs of *Geobacter* sp., the ImcH homologs of *Geobacter* sp. were also clustered together as well as with those from other bacteria, such as *Desulfuromonas* sp. and *Syntrophus* sp. (Figure 6). The identities among these homologs were 53.6–100%, also suggesting a conserved functional role among these homologs. The identities between this group of ImcH homologs and those from other bacteria were 31.5–49%. The lengths of polypeptides of ImcH homologs identified from *Geobacter* sp. and their associated homologs ranged from 495 to 519 amino acid residues. They all contained 3 transmembrane motifs at their *N*-terminal part, an atypical CX_3_CH motif next to the transmembrane domain and six typical CX_2_CH motifs (Figure 6 and Supplementary Table S3). Similar to the ImcH homologs of *Geobacter* sp., the ImcH homologs identified from other bacteria all had 3 transmembrane motifs and an atypical CX_3_CH motif, except for the ones of *Intrasporangium calvum* and *Anaerolinea thermophila* which contained only one transmembrane motif (Figure 6 and Supplementary Table S3). Moreover, the CX_2_CH motifs of identified ImcH homologs varied, ranging from 5 to 9 (Figure 6 and Supplementary Table S3). Similar to *cymA* and *cbcL*/*mtrH*/*mtoC, imcH* genes were also often associated with the genes encoding *c*-Cyts. Different from *cymA* and *cbcL*/*mtrH*/*mtoC*, however, no *mtrAB*/*mtoAB* were found to be adjacent to *imcH* (Figure 7).

Previous results showed that ImcH was found in other bacteria with EET capability, including several *Acidobacteria* sp. and *Geobacter* sp. as well as *Anaeromyxobacter dehalogenans* 2CP-1, *G. ferrihydriticus*, *G. subterraneus*, *Geopsychrobacter electrodiphilus, Geothrix fermentans*, *M. roseus*, and *Pelobacterselenii genes*, suggesting a broad involvement of ImcH in EET (Levar et al., 2014). However, all of these bacteria transfer electrons from cytoplasmic membrane to extracellular electron acceptors. If they are involved in EET, the ImcHs of these bacteria would function as quinol oxidases. Search results from this study did not identify additional ImcH from the bacteria with EET capability. Thus, different from CymA and CbcL/MtrH/MtoC that are implicated in both quinol oxidation and quinone reduction, ImcH has only been implicated in quinol oxidation so far.

Previous results also showed that the homologs of CymA and CbcL/MtrH/MtoC families or the homologs of CbcL/MtrH/MtoC and ImcH families could be found in the same bacterium (Shi et al., 2012; Levar et al., 2014; Zacharoff et al., 2015). In *G. sulfurreducens*, CbcL and ImcH participate in EET to the terminal electron acceptors with different redox potential (Levar et al., 2014, 2017; Zacharoff et al., 2015). Results from this study demonstrate that most *Geobacter* sp. investigated as well as other metal-reducing bacteria also have both CbcL and ImcH, where they may participate in EET to the terminal electron acceptors with different redox potentials. Moreover, in the metal-reducing bacterium *R. ferrireducens*, 2 *cymAs* and a *mtrH* are in the same gene cluster that also has *mtrABC* and the genes encoding for other *c*-Cyts. The two CymAs of *R. ferrireducens* are only 55.5% identical, which suggests that these two CymAs may oxidize different quinols. Thus, it is possible the CymAs and MtrH of *R. ferrireducens* are also involved in regulating EET to different terminal electron acceptors. Possessions of different quinol oxidases for EET would render the microorganisms better adapted to the changing environment. Finally, it should also be noted that homologs of CymA, CbcL/MtrH/MtoC, and ImcH families are not found in the same microorganism in this study.

## Conclusion

Crucial to bacterial and probably archaeal EET are the quinol oxidases and quinone reductases in the cytoplasmic membrane. These groups of enzymes function either as the first step during electron transfer from the cytoplasmic membrane to extracellular electron acceptors and/or the last step during electron transfer from extracellular electron donors to the cytoplasmic membrane. CymA, CbcL/MtrH/MtoC and ImcH were the only three protein families that were known to be involved in these crucial EET steps during this search. Survey of bacterial genomes from this study reveals that CymA family is only restricted to the phylum of Proteobacteria and most of the homologs are found in the *Shewanella* genus, while CbcL/MtrH/MtoC and ImcH protein family are much more widespread among different bacterial phyla, demonstrating much broader distributions of latter two protein families in bacterial EET.

A unique feature for bacterial EET is its bidirectional nature (Shi et al., 2012). This is reflected by the involvements of Mtr homologs in both Fe(II) oxidation and Fe(III) reduction (Beliaev and Saffarini, 1998; Jiao and Newman, 2007), quinol oxidation and quinone reduction capability of CymA (Marritt et al., 2012a; McMillan et al., 2013) and reversible conductivity with nearly equal efficiency of MtrF and its homologs, such as MtrC (Breuer et al., 2012, 2014). Indeed, *S. oneidensis* MR-1 employs the Mtr pathway for transferring electron from the cytoplasmic membrane to electrode surfaces as well as from electrode surfaces to the cytoplasmic membrane under anoxic condition (Ross et al., 2011). Identification of CbcL/MtrH/MtoC homologs in both Fe(II)-oxidizing and Fe(III)-reducing bacteria are consistent with the bidirectional nature of bacterial EET. However, ImcH homologs have been identified only in the Fe(III)-reducing bacteria to date. The reason for this observation is currently unknown. It may be attributed to limited homologs identified, lack of insightful understanding of bacterial Fe(II) oxidation process or inability to reduce quinone by ImcH.

Lack of apparently distinctive feature between CbcL/MtrH homologs and MtoC homologs also indicate that the direction of catalysis and electron transfer of CbcL/MtrH/MtoC may be regulated by protein–protein interactions. Indeed, CbcL, MtoC and probably MtrH need to interact with other periplasmic redox proteins, such as *c*-Cyts, in order to exchange electrons with extracellular substrates (Shi et al., 2012, 2016). Previous results also demonstrated the importance of biochemical characterization of CymA from *S. oneidensis* MR-1 in understanding the underlying mechanisms of this type of redox enzymes for regulating their catalytic activity and electron transfer reactions. Thus, biochemical characterization of CbcL and ImcH homologs will be one of the future directions for investigating the catalytic properties of these new families of redox enzymes crucial for bacterial EET.

## Author Contributions

LS designed the experiments. YZ performed the experiments. YZ and LS analyzed the data and prepared the manuscript.

## Conflict of Interest Statement

The authors declare that the research was conducted in the absence of any commercial or financial relationships that could be construed as a potential conflict of interest.
